# Motivating Cord Blood Donation with Information and Behavioral Nudges

**DOI:** 10.1038/s41598-017-18679-y

**Published:** 2018-01-10

**Authors:** Daniela Grieco, Nicola Lacetera, Mario Macis, Daniela Di Martino

**Affiliations:** 1 Università Bocconi, Milan, Italy; 2 University of Toronto, Toronto, Canada; 3 Johns Hopkins University, Baltimore, USA; 4 Department of Woman, Mother, and Neonate, Buzzi Children’s Hospital, Biological and Clinical Sciences, University of Milan, Milan, Italy

## Abstract

Umbilical cord blood is a source of hematopoietic stem cells essential to treat life-threatening diseases, such as leukemia and lymphoma. However, only a very small percentage of parents donate upon delivery. The decision to donate the cord blood occurs at a very specific time and when parents likely experience emotional, informational, and decisional overloads; these features of cord blood donation make it different from other pro-social activities. In collaboration with an OB-GYN clinic in Milan, Italy, we conducted the first randomized controlled trial that applies tools from behavioral science to foster cord blood donation, and quantified the gains that informational and behavioral “nudges” can achieve. We found that information and “soft” commitments increased donations; approaching expecting parents closer to the delivery date and providing them with multiple reminders, moreover, had the strongest impact. However, a significant portion of women who expressed consent to donate could not do so because of organizational constraints. We conclude that simple, non-invasive behavioral interventions that address information gaps and procrastination, and that increase the salience of the activity can substantially enhance altruistic donations of cord blood, especially when coupled with organizational support.

## Introduction

Up to one in three individuals may benefit from regenerative medicine therapy through stem cells^1^. Together with bone marrow and peripheral blood stem cells, umbilical cord blood is a source of hematopoietic stem cells for children and adults who face life-threatening diseases and need a transplant of blood-forming cells^2^. A primary use of cord blood stem cells is to treat different types of hematological malignancy in children. Cord-blood-derived stem cells are also used to treat such diseases as acute and chronic leukemia, lymphoma, solid tumors, thalassemia, sickle cell disease and severe aplastic anemia. Stem cells also are expected to be used in the future to treat spinal cord injury, cerebral palsy, diabetes, heart disease, stroke and Parkinson’s disease. Their potential uses are promising and far from being fully explored.

Extracting cord blood is a fast, painless procedure performed during delivery (see the Supplementary Material for further information). In the case of donation to a public bank, it does not entail financial costs for the donor. Because cord blood units that have been stored are ready to use, cord blood donations are particularly useful for recipients who need an urgent transplant. Also, the transplant of cord blood stem cells is less likely to cause rejection or graft-versus-host disease compared to bone marrow or peripheral blood stem cells^3,4^. Finally, the use of cord blood stem cells does not present ethical or religious controversies, unlike, for example, stem cells from embryos.

These features, combined with the fact that over 130 million babies are born each year worldwide, make the collection of cord blood a promising and efficient route to address many serious illnesses and to advance medical research^4^. However, only a small share of parents opt to donate the cord blood upon delivery of a baby. Some parents choose to store it in a private bank for self-use, for a fee, although presently there is no strong evidence of the benefits of self-transplants. In the vast majority of cases, cord blood is simply discarded as medical waste (more than 95% of the time in the United States)^5^. In Italy, about 1% of parents donate the cord blood to a public cord blood bank. Donations are needed also to ensure more genetically diverse inventories, with better matches and higher survival rates as a result^6,7^.

In this paper, we present the results of a randomized field experiment in which we applied theories and methods from behavioral science to study the motivations for and impediments to umbilical cord blood donation by expecting parents. Although there is an emerging body of medical and public-health literature about the donation and conservation of cord blood^4,8^, little research is available in the social sciences. In fact, to our knowledge this is the first field-experimental study to apply ideas from behavioral economics in the context of donations of umbilical cord blood. Because the decision to donate cord blood presents specificities that make it different even from similar prosocial activities for which there is now a broader literature (e.g., giving whole blood), studies focused on cord blood donation allow academics, practitioners, and policymakers to explore specific behavioral mechanisms that potentially could be used to recommend specific interventions or “nudges^9^”.

There are several reasons for the very low cord blood donation rates currently observed. At a basic level, because of the relative novelty of this donation possibility, people may lack the relevant information. In addition, cord blood donation can only occur at a very specific time: i.e., during delivery. Thus, unlike with other types of donations, individuals have much less discretion about when to donate; failure to decide at the “right time” will eliminate the possibility of donating. Also, this specific donation moment coincides with potential donors being overwhelmed by numerous decisions as well as experiencing informational and emotional burden. Evidence of these forms of overload is present for health-related situations that, although clearly different, present similar features, such as organ donation decisions by next-of-kin of potential deceased donors^10^ or end-of-life decisions^11^. Finally, the donation decision is not dichotomous because parents have three options: doing nothing, donating to a public bank, or paying for self-storage at private cord blood banks for possible future use. However, self-storage of cord blood is prohibited in Italy, although some parents opt to store it in private blood banks abroad (to date, however, there is no strong evidence for benefits of privately stored cord blood)^2^.

Organizations and public agencies interested in enhancing cord blood donations thus face peculiar challenges and opportunities, and insights from behavioral science offer tools to address them. To start, simply providing information about an activity that has been much less promoted than others might offer enough stimuli to increase donations. Moreover, timing issues might be crucial given that donations cannot be made at any time; therefore, precisely when information is provided to the expecting parents, and when these individuals are asked to express their intention or make a decision, is likely to matter. For example, if someone is asked early in pregnancy, the temporal distance from the actual event might lead to both cognitive and emotional distance^12–14^, such that individuals might not fully appreciate the implications of their choice. Issues such as self-control and procrastination may also be relevant^15–17^; at a certain point during the pregnancy, parents may agree to donate the cord blood upon delivery, but they might change their minds or simply overlook this issue as the delivery date approaches and they are preoccupied with other issues that require immediate attention. Behavioral investigations found that facing an endeavor (such as making a choice, taking on a responsibility, or resisting a temptation) might undermine self-regulation in a subsequent, unrelated domain^18^. In the context of informational and emotional overload, salience may also play a relevant role^19,20^. The decision to donate cord blood thus shares similarities with a number of other contexts in which information, salience, self-control, and overload might significantly affect decisions, as in the case of financial saving decisions, choosing among insurance options, or undertaking certain medical tests^21–28^.

We conducted our randomized controlled experiment in collaboration with the OB-GYN clinic of the Ospedale dei Bambini Vittore Buzzi (Buzzi Hospital or BH hereafter) in Milano, Italy, between September 2014 and early June 2016. We considered the peculiarities of the cord blood donation decision described above to define our treatment conditions, based on variation in the provision of information and the possibility for the participants to express a “soft” (non-binding) commitment^29^ to donate, combined with variations in the timing and frequency at which expecting parents were treated (the details are in the Methods section below). The variation in the timing of the interventions allowed us to explore the tension between salience and psychological overburden. The option to express a soft commitment allowed us to test, in a context where choice cannot be forced or even made binding, whether a direct expression of interest can complement the provision of information in motivating certain prosocial actions. A mechanism that could be at play in this context is that expecting parent(s) might incur a psychological cost, such as guilt or poor self-esteem for not making the donation after having expressed their intention to do so^2,30^.

The effectiveness of these simple and minimally invasive behavioral treatments or nudges, however, may also depend on the organizational complexity around the activity that one wants to promote. When the behavior of interest concerns only the directly interested individual (e.g., exercising, eating healthy foods, smoking cessation, saving for retirement), interventions that successfully influence their decisions are plausibly sufficient to produce the desired results. In other cases, however, the performance of a given activity involves the intervention of third parties, say because of the required expertise, the inherent complexity of the activity, or organizational rules. The donation of umbilical cord blood falls in this category. The 2006 Scientific Report of the Royal College of Obstetricians and Gynecologists, for example, identifies several procedural and organizational issues related to the donation, collection, and storing of umbilical cord blood: the consent procedure and associated paperwork (before and after collection and storage) place an additional load on an already overstretched midwifery staff; the collection procedure must occur at a time when both mother and baby require one-on-one care; the use of midwifery or medical staff for cord blood collection may distract them from the care of other mothers and babies^31^. According to the national legislation of relevance, moreover, hospital staff such as obstetric nurses need to obtain certification in order to extract and process the cord blood. This is the case in Italy, for example. The transfer of the blood unit to a cord blood bank, in turn, depends on regulations and on additional personnel, and on coordination between the hospital and the public cord blood bank. In general, therefore, collection and storage of cord blood impose considerable logistical burdens on obstetricians, midwives, and the hospital, and might be difficult to carry out in moments of intense overload in the delivery room. In such a case, therefore, although behavioral treatments might stimulate the start of the process with individuals expressing their interest and consent, the remaining phases include a multiplicity of people, organizations and rules.

Our design included six experimental conditions differing on the following elements: (a) the provision of additional information about cord blood donation, (b) the possibility of expressing a non-binding “prompted choice” to donate, and (c) the timing of the intervention (1st trimester, 3rd trimester, or both). The outcomes we consider include the *stated* expression of interest in donating cord blood, actions that indicate the *actual intention to donate* (e.g., submitting signed inform consent forms for donation), and *actual donations* of cord blood. Moreover, we also analyze how organizational constraints inhibit the potential effectiveness of information and behavioral nudges; this is a relevant issue that, so far, has received only limited attention.

## Results

**Figure 1:**
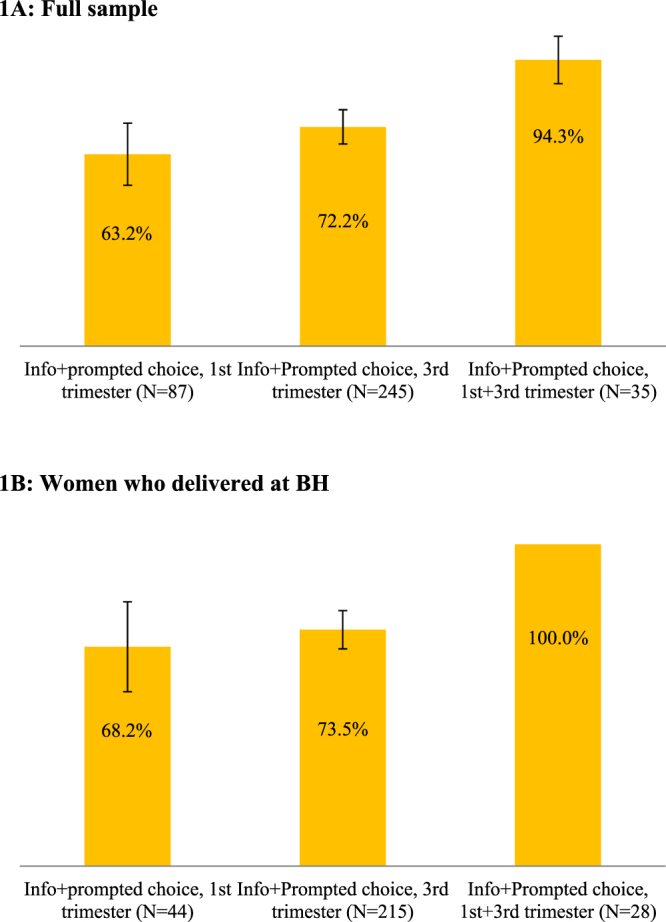
Share of women who reported intention to donate. The samples include women in treatment groups that included the “prompted choice” feature (T2, T4, and T5). Error bars represent 95% confidence intervals (±1.96*s.*e*.).

**Figure 2:**
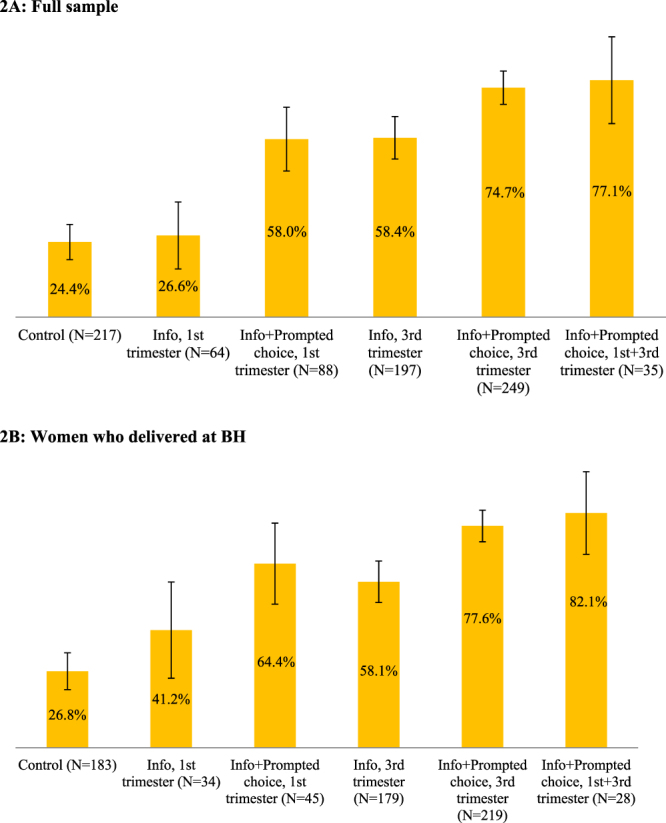
Share of women who requested the donation forms. Error bars represent 95% confidence intervals (±1.96*s.*e*.).

**Figure 3:**
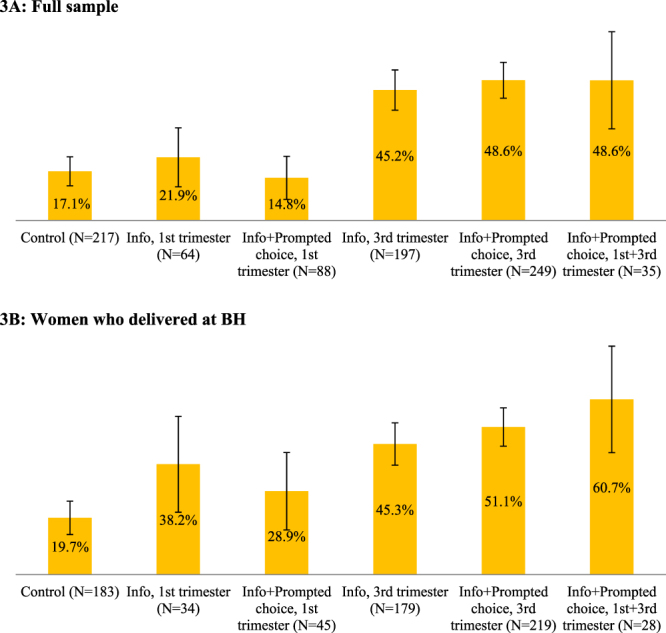
Share of women who submitted their signed donation consent forms. Error bars represent 95% confidence intervals (±1.96*s.*e*.).

**Figure 4:**
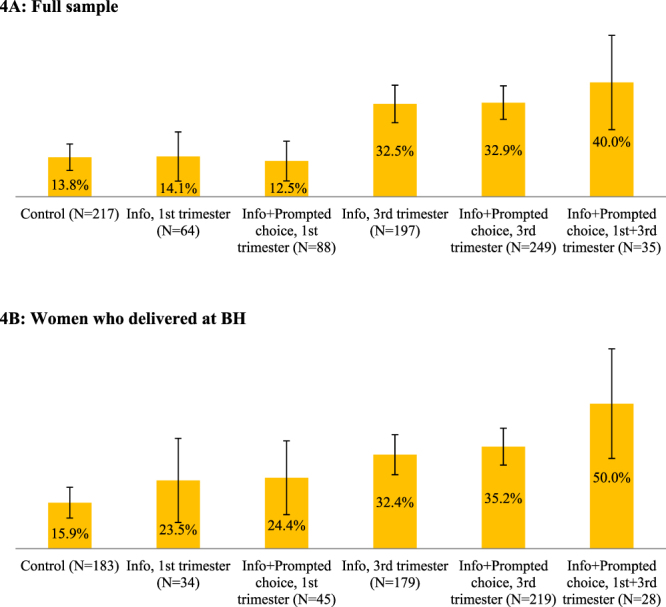
Share of women who submitted their signed donation consent forms and were eligible to donate. Error bars represent 95% confidence intervals (±1.96*s.*e*.).

**Figure 5:**
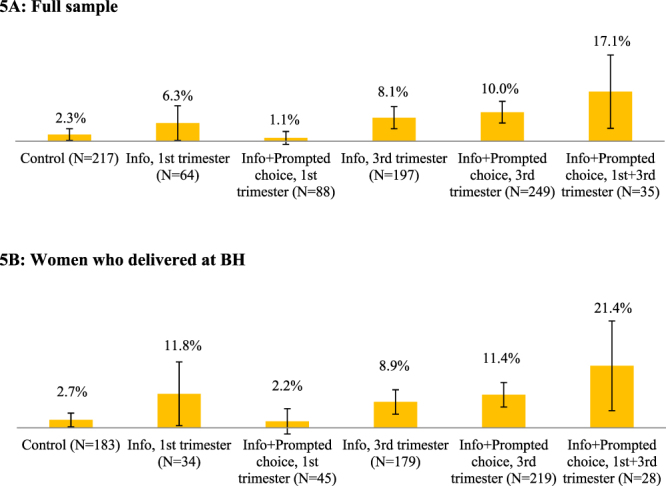
Share of actual cord blood donations. Error bars represent 95% confidence intervals (±1.96*s.*e*.).

**Figure 6:**
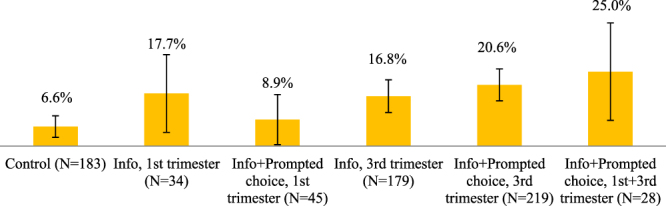
Potential donation rates in the absence of organizational and institutional constraints. The figure shows the shares of women who submitted their signed donation consent forms, were eligible to donate, delivered at BH, and did not present complications during delivery. Error bars represent 95% confidence intervals (±1.96*s.*e*.).

We performed our analyses on the full sample of participants as well as on the sample of women who gave birth at BH. The advantage of the latter sample is that we have full information about these participants’ donation intentions and behavior. Results from the full sample are conservative because we assume that all women for whom we do not observe outcomes have not requested forms or donated, although some of them might have done it elsewhere. We report the raw means in Figs 1–6 below. To test for the effects of our treatments on the outcomes of interest and their statistical significance, we estimated Ordinary Least Squares regressions (results in Tables 1 and 2) separately for the full sample (Table 1) and for the sample of women who delivered at BH (Table 2). The top panels of each table report the estimated treatment effects (i.e., the differences in the average outcomes of each condition from the control group) whereas the bottom panels show the estimated pairwise differences of effects between the five treatment conditions. In the Supplementary Material, we also report the treatment effect estimates from regressions that include control variables derived from administrative records and from a survey that we conducted (Tables S4 and S5). The results are very similar to those reported here.

**Table 1:** Treatment effects – Full Sample.

	Outcome variable				
	Expressed intention to donate	Requested donation info and consent forms	Handed in signed donation consent forms	Handed in signed donation consent forms, and eligible to donate	Actual donation
**Treatment condition**					
Control group (constant in the regression)		0.244**	0.171**	0.138**	0.023
		(0.031)	(0.031)	(0.029)	(0.017)
Info, 1st trimester		0.021	0.048	0.002	0.039
		(0.065)	(0.064)	(0.060)	(0.035)
Info + Prompted choice, 1st trimester	0.632**	0.335**	−0.023	−0.013	−0.012
	(0.047)	(0.058)	(0.057)	(0.053)	(0.031)
Info, 3rd trimester		0.340**	0.281**	0.187**	0.058*
		(0.045)	(0.044)	(0.041)	(0.024)
Info + Prompted choice, 3rd trimester	0.090	0.503**	0.315**	0.191**	0.077**
	(0.055)	(0.042)	(0.042)	(0.039)	(0.023)
Info + Prompted choice, Multiple ask	0.311**	0.527**	0.315**	0.262**	0.148**
	(0.089)	(0.083)	(0.082)	(0.077)	(0.045)
N. of observations	367	850	850	850	850
R squared	0.033	0.173	0.099	0.053	0.026
**Tests of differences between estimated parameters**					
(Info + Prompted choice 1st trim) − (Info 1st trim)		0.314**	−0.071	−0.016	−0.051
		(0.075)	(0.074)	(0.069)	(0.041)
(Info 3rd trim) − (Info 1st trim)		0.318**	0.233**	0.184**	0.019
		(0.066)	(0.065)	(0.061)	(0.036)
(Info + Prompted choice 3rd trim) − (Info + Prompted choice 1st trim)		0.168**	0.338**	0.204**	0.089**
		(0.056)	(0.056)	(0.052)	(0.031)
(Info + Prompted choice 3rd trim) − (Info 3rd trim)		0.163**	0.034	0.004	0.019
		(0.043)	(0.043)	(0.040)	(0.024)
(Info + Prompted choice, Multiple ask) − (Info + Prompted choice 3rd trim)	0.220**	0.024	0.000	0.071	0.071
	(0.080)	(0.082)	(0.082)	(0.076)	(0.045)
(Info + Prompted choice, Multiple ask) − (Info + Prompted choice 1st trim)		0.192*	0.338	0.275**	0.160
		0.091	(0.090)	(0.084)	(0.050)

Notes: Ordinary least squares regressions. Standard errors are in parentheses. **p < 0.01, *p < 0.05, ^+^ *p* < 0.1.

**Table 2:** Treatment effects – Women who delivered at BH.

	Outcome variable					
	Expressed intention to donate	Requested donation info and consent forms	Handed in signed donation consent forms	Handed in signed donation consent forms, and eligible to donate	Handed in signed donation consent forms, eligible to donate, and no complications during delivery	Actual Donation
**Treatment conditions**						
Control group (constant in the regression)		0.268**	0.197**	0.158**	0.066*	0.027
		(0.034)	(0.035)	(0.033)	(0.026)	(0.020)
Info, 1st trimester		0.144^+^	0.186*	0.077	0.111^+^	0.090^+^
		(0.085)	(0.088)	(0.083)	(0.066)	(0.051)
Info + Prompted choice, 1st trimester	0.682**	0.377**	0.092	0.086	0.023	−0.005
	(0.064)	(0.075)	(0.079)	(0.074)	(0.059)	(0.045)
Info, 3rd trimester		0.313**	0.256**	0.166**	0.102**	0.062*
		(0.048)	(0.050)	(0.047)	(0.037)	(0.029)
Info + Prompted choice, 3rd trimester	0.053	0.508**	0.315**	0.193**	0.140**	0.087**
	(0.070)	(0.045)	(0.047)	(0.045)	(0.036)	(0.027)
Info + Prompted choice, Multiple ask	0.318**	0.554**	0.410**	0.342**	0.184*	0.187**
	(0.103)	(0.092)	(0.096)	(0.090)	(0.072)	(0.055)
N. of observations	287	688	688	688	688	688
R squared	0.037	0.171	0.076	0.040	0.028	0.028
**Differences between treatment conditions**						
(Info + Prompted choice 1st trim) − (Info 1st trim)		0.233*	−0.093	0.009	−0.088	−0.095
		(0.103)	(0.107)	(0.101)	(0.081)	(0.062)
(Info 3rd trim) − (Info 1st trim)		0.169*	0.070	0.089	−0.009	−0.028
		(0.085)	(0.088)	(0.083)	(0.066)	(0.051)
(Info+Prompted choice 3rd trim) − (Info+Prompted choice 1st trim)		0.132^+^	0.223**	0.107	0.117*	0.091*
		(0.074)	(0.077)	(0.073)	(0.058)	(0.045)
(Info+Prompted choice 3rd trim) − (Info 3rd trim)		0.195**	0.059	0.028	0.038	0.025
		(0.046)	(0.048)	(0.045)	(0.036)	(0.028)
(Info + Prompted choice, Multiple ask) − (Info + Prompted choice 3rd trim)	0.265**	0.045	0.096	0.148^+^	0.045	0.100^+^
	(0.086)	(0.091)	(0.095)	(0.089)	(0.071)	(0.055)
(Info + Prompted choice, Multiple ask) − (Info + Prompted choice 1st trim)		0.177	0.318**	0.256*	0.161^+^	0.192**
		(0.109)	(0.114)	(0.107)	(0.085)	(0.066)

Notes: Ordinary least squares regressions. Standard errors are in parentheses. **p < 0.01, *p < 0.05, ^+^*p* < 0.1.

### Intention to donate

As shown in Fig. 1A, a large proportion of women in the “prompted choice” conditions (T2, T4, and T5) indicated that they intended to donate the cord blood to the local public bank: 63.2% in T2 (55/87), 72.2% in T4 (177/245), and 94.3% (33/35) in T5. Of the 35 women in condition T5, 60% (21/35) expressed their interest when first asked in the first trimester, and 34.3% (12/35) of the remaining women who did not express interest in the first trimester did express it when asked again; conversely, no women who expressed interest in the first trimester reverted their “prompted choice” later in the pregnancy. In Fig. 1B, we report the proportions of women expressing intention to donate while restricting the sample to the 287 women in treatments T2, T4, and T5 who delivered at the BH; the share expressing interest in donating were 68.1% (33/44) in T2, 73.5% (158/215) in T4, and 100% (28/28) in T5.

Column 1 of Tables 1 and 2 show that the effect of information plus prompted choice treatment was statistically indistinguishable for women approached in the 1^st^ and 3^rd^ trimesters; by contrast, the higher effect on women who were “asked twice” was statistically different from conditions T2 and T4 (*p* < 0.01).

### Requesting consent and medical forms

In Fig. 2, we report the shares of women who requested consent and medical history forms, again in the full sample (2 A) and in the sample limited to women who delivered at BH (2B). Twenty-four percent of the women in the control group (53/217) requested the consent and medical history forms compared to 26.6% in T1 (17/64), 58.0% in T2 (51/88), 58.4% in T3 (115/197), 74.7% in T4 (186/249), and 77.1% in T5 (27/35). With the exception of group T1 (i.e., information only in the 1^st^ trimester), the positive treatment effects of the other experimental conditions with respect to the control group were statistically significant, as shown in Column 2 of Table 1 (p < 0.01 in all cases). Moreover, the prompted choice opportunities were significantly more effective than providing only information in the same trimester (*p* < 0.01), and that the effect of providing only information in the third trimester was significantly higher than providing it in the first trimester (*p* < 0.01). The T5 group (multiple presentations of information with prompted choice) caused the highest rate of request of consent material, although this rate was not statistically different from that of women in T4 who received information and a prompted choice opportunity only late in the pregnancy.

For the 688 women who delivered at BH, the forms were requested by 26.8% of those in the control group, 41.2% in T1, 64.4% in T2, 58.1% in T3, 77.6% in T4, and 82.1% in T5 (Fig. 2B). The regression results from Table 2 (column 2) display similar statistical significance for the differences from the control and between treatment conditions although, in general, these estimated differences are smaller in magnitude and have larger standard errors.

### Submitting completed and signed consent and medical forms

In this study, the submission of the consent and medical forms is the most relevant outcome to assess the behavioral impact of our interventions and to determine the potential role of different psychological mechanisms in determining willingness to donate. The shares of women who directly expressed their willingness to donate cord blood to the local public bank by signing and handing in the consent and medical history forms provided by the hospital (Fig. 3A) were 17.1% in the control group (37/217), 21.9% in T1 (14/64), 14.8% in T2 (13/88), 45.2% in T3 (89/197), 48.67% in T4 (121/249) and 48.6% in T5 (17/35). Only the effects of treatments occurring in the third trimester of a pregnancy (T3, T4, and T5) were statistically different from the control (Table 1, Column 3), and the effects of T3 and T4 were statistically different from the corresponding early-pregnancy conditions T1 and T2; however, none of the effects of late pregnancy interventions were statistically different from each other.

Among those who delivered at BH (Fig. 3B), the shares of women who submitted their signed medical and consent forms were slightly higher than in the full sample: 19.7% in the control group, 38.2% in T1, 28.9% in T2, 45.3% in T3, 51.1% in T4, and 60.7% in T5. The estimated treatment effects (Table 2, Column 3) are broadly similar to those for the full sample, with the exception that the effect of providing information only in the 1^st^ trimester was statistically significant compared to that of the control group (*p* < 0.05). More generally, for women in early pregnancy who delivered at BH the treatment effects were larger than the early-pregnancy treatments for the whole sample.

### Excluding participants who expressed consent but were ineligible to donate

A non-negligible share of women who formally consented to donate was not, however, eligible to do so. Once we exclude ineligible women (from the numerator but not the denominator), the shares of those who committed to donate become 13.8% in the control group, and 14.1%, 12.5%, 32.5%, 32.9%, and 40% T1, T2, T3, T4, and T5, respectively (Fig. 4A). Again, the effects of late-pregnancy treatments were statistically different from the control (Table 1, Column 4), and the effects of T3 and T4 were statistically different from T1 and T2. The corresponding shares among women who delivered at BH were 12.9%, 23.5%, 24.4%, 32.4, 35.2%, and 50% (Fig. 4B) with rates for late pregnancy interventions being statistically different from the rate for the control (Table 2, Column 4).

The increased salience of information and soft commitment options given later in the pregnancy, therefore, attracted a relatively high share of women who would not be eligible to donate. However, the overall share of eligible donors remained larger in late-pregnancy treatments than in the control and early pregnancy groups.

### Actual cord blood donations

Figure 5 reports the proportions of women in the various experimental conditions who successfully donated the umbilical cord blood upon delivering their baby at BH. The rate in the control group, 2.3% (5/217), was close to the historical donation rate at BH. In the treatment conditions, successful donations occurred for 6.3% (4/64) of women in T1, 1.1% (1/88) in T2, 8.1% (16/197) in T3, 10.0% (25/249) in T4, and 17.1% in T5 (6/35), for a total of 57 cord blood units collected (Fig. 5A). The corresponding rates among women who delivered at BH (Fig. 5B) were 2.7% (5/183) in the control group, 11.7% (4/34) of women in T1, 2.2% (1/45) in T2, 8.9% (16/179) in T3, 11.4% (25/42) in T4, and 21.4% (6/28) in T5. In both samples that we analyze, the treatment effects were significantly greater than zero for the late-pregnancy conditions. Although the smaller number of observations led to larger standard errors, the size of the differential impact of the multiple-inquiry treatment T5 is substantial.

### Reasons for unsuccessful donations

Of the 197 women who submitted the consent forms, were medically eligible to donate, and delivered at BH (i.e., the total pool of potential donations), 57 successfully donated whereas 140 did not. We have detailed information about the reasons for missed donations on 109 of the 140 cases: 62 (56.9%) could not donate because of medical complications during the delivery, 33 (30.3%) because of organizational reasons including overcrowding of the delivery room and the absence of obstetric nurses certified to collect and process cord blood at the time of the delivery, and 14 (7.1%) potential donations did not occur for institution-related reasons: for example, because the delivery occurred outside the opening hours of the Milan Cord Blood Bank.

### Potential number of donations in the absence of organizational and institutional constraints

In Fig. 6, we report the shares of potential donations. In each experimental condition, the denominator includes all women in the study who delivered at BH, and the numerator comprises women who submitted their consent and medical forms, were eligible to donate, and did not present complications during delivery. That is, we show what donation rates would have been if organizational and institutional impediments had been absent. Donations rates would have been 6.6% (12/183) in the control, 17.7% (6/34) in T1, 8.89% (4/45) in T2, 16.8% (30/179) in T3, 20.6% (45/219) in T4, and 25% (7/28) in T6, with the higher shares in the late-pregnancy treatments being statistically different from the control, and, in some cases (T4 and T5 vs. T2), from the early-pregnancy treatment groups (Table 1, Column 5).

## Discussion

The first-order insight from this study is that our interventions, although mild and not implying any binding choice, were nonetheless effective in increasing future parents’ interest in donating, their willingness to take further actions to make the donation possible, and, eventually, actual donations. Lack of information and limited attention (which can be enhanced through the expression of a soft commitment) thus help explain the current low donation rates. The timing of the decision was of first-order importance; the evidence shows that approaching the expecting parents closer in time to the relevant action had a major impact. The prompted choice request was particularly effective when administered in the third trimester. Procrastination thus appears to be a major barrier to the cord blood donation decision, and providing more salience to this activity closer to the natural deadline increased both expressions of interest and actual donations. The increased salience of information and soft commitment options given later in the pregnancy, however, also induced a relatively higher share of women who were later determined to be ineligible. Nonetheless, the share of eligible donors generated by the prompted choice treatment remained considerably larger than in the control and early pregnancy treatment conditions. Moreover, repeated asks, which can function as reminders, were effective. Of particular interest in the case of the multiple ask was the fact that, although asking again led some women who had not expressed willingness to donate in the first term to do so later in the pregnancy, none of the participants reversed an early expression of interest. This finding is similar to what has been documented in previous studies: for example, with reference to multiple asks to join the organ donor registry^10^.

Overall, therefore, motivating donations through behavioral “nudges” is promising according to our results. Compared to the control group, which presented a baseline of 2.3% donation rate in the full sample and 2.7% among those participants who delivered at BH, information and soft commitment opportunities administered in the 3^rd^ trimester increased actual donations by a statistically significant 5.8–6.2 and 7.7–8.7 percentage points, respectively, and multiple asks by 14.8–18.7 percentage points (the first figure refers to the full sample and the second to the sample restricted to BH deliveries). The parents in our sample made a total of 57 successful cord blood donations; taking the actual donation rates of the control group as the default, in the absence of this intervention, the total number of donations would have been between 18 and 20.

At the same time, there was a large gap between the pool of women who submitted signed donation consent forms and actual donations. Although most of this gap was due to “exogenous” causes such as medical ineligibility and complications during delivery, a significant portion of missed donations was due to organizational and institutional features of the hospital and the blood banking system. Our findings imply that the units of umbilical cord blood collected could have almost doubled (from 57 to 104) with additional delivery rooms, licensed obstetric nurses, and more extended opening hours at the local public cord blood bank. As hypothesized, the donation of umbilical cord blood, because of the complexity of the consent and especially of the collection and storage procedures, would be enhanced not only through simple behavioral interventions but also through organizational improvements to support the will of expecting parents. Naturally, removing organizational and institutional constraints can be costly. However, it is important to have a sense of the gains that could be achieved with their removal, and our paper is the first to quantify these gains.

A limitation of our study is that the sample sizes of our treatment groups were unbalanced, with fewer participants in the first trimester. In spite of the reduced statistical power for those experimental conditions, the point estimates were generally small, thus attenuating concerns related to sample size. Another limitation is that attrition from the sample disproportionally reduced the size of the “multiple ask” treatment group; however, the size of the treatment effects in this group was large enough that we were still able to detect them with statistical precision.

Moreover, although we draw policy implications from our findings, the results could be specific to the context that we studied and might not entirely extend to other settings. In this respect, we hope that future studies will replicate our design in other contexts. For example, in countries where self-storage in private cord blood banks is legal (e.g., the United States), the relevant choice set that parents have is different than in Italy. The easier availability of a third, self-storage alternative may lead to different effects of the same behavioral interventions, and enable exploration of other interesting psychological mechanisms such as the role of risk preferences.

In conclusion, the results from our novel trial indicate that simple, non-invasive behavioral interventions can substantially enhance altruistic donations of cord blood, particularly when accompanied by adequate organizational and institutional support. We believe that our results can provide guidance to clinics and cord blood banks and thus contribute to increasing the supply of this life-saving resource and to the broader literature on the role of ideas and methods from behavioral science in health-related settings^32,33^.

## Methods

### Ethics approval

This project was reviewed and approved by the Research Ethics Board of the University of Toronto (Protocol 30487) and the Homewood Institutional Review Board of Johns Hopkins University (Protocol 2183), and received clearance from the Ospedale Buzzi. All methods were performed in accordance with the relevant guidelines and regulations.

### Setting: Cord blood donation in Italy and the “Ospedale dei Bambini Vittore Buzzi”

A 2009 law regulates the use of cord blood in Italy and its donation for the benefit of unrelated patients. Public cord blood banks manage the collection and storage of cord blood, free of charge to the donor^3^. After a donation, the blood is tested at the blood bank to determine whether the quantity collected was sufficient (the cord blood is banked if the quantity of blood is at least 60 milliliters) and whether the quality is such that the unit can be preserved. Self-storage (also in public banks) is allowed and free if certain medical conditions are met^3^; if there are no clinical reasons to justify it, self-storage can be done only in a cord blood bank located abroad, and the donor is responsible for the cost^3^.

We conducted our study in collaboration with the OB-GYN clinic of a hospital in Milano, Italy: the “Ospedale dei Bambini Vittore Buzzi” (BH). BH hosts the second largest maternity ward in the city, with about 3,500 deliveries per year. The cord blood collected is stored in the “Milano Cord Blood Bank” (MCBB)^34^, which was established in 1993 and has branches in forty hospitals in the Lombardy region (of which Milano is the capital and largest city) and in the province of Trento, in Northern Italy. Throughout its activity, the MCBB collected more than 31,000 cord blood units. Because of the quantity and quality medical requirements, only about one third (9,921 units) have been cryo-preserved and stored. With a total of about 25,000 units stored in the 19 regional cord blood banks active in Italy, the MCBB is the largest in the country.

As part of their standard operations, MCBB personnel at BH provide some information to expecting parents about medical, ethical and legal aspects of cord blood donation. If interested, parents declare their consent after completing a detailed anamnestic questionnaire to evaluate the general health status of the mother and the presence of risk factors from either parent. The informed consent implies: (*i*) the registration of the donor’s personal data; (*ii*) the cord blood collection and conservation; (*iii*) the possibility of using the cord blood for transplants (or for research aims if a transplant cannot be executed); (*iv*) the perspective mother’s permission to be contacted after six months from the delivery to check on the baby’s health status.

Obstetric nurses are the staff mostly in charge of the procedure at BH; they meet the expecting mothers during their routine medical visits or the pre-delivery courses, distribute questionnaires and consent forms, and collect them.

Women who plan to give birth at BH visit the clinic in at least two occasions. During the first trimester, a visit is scheduled around the 12^th^ week to perform blood tests as well as a nuchal translucency ultrasound. During the third trimester, women visit the clinic for an “end-of pregnancy” checkup (at a separate ambulatory facility) around the 36–38^th^ week of gestation. Women may receive additional care during the pregnancy at the clinic, but many instead consult physicians in other clinics or private practices for additional visits.

Cord blood donations at BH have historically been very low (between 1% and 3%); in 2011, the hospital made it a strategic priority to increase them. With our interventions, we are contributing to this objective.

### Experimental conditions

Our treatment conditions included (a) providing the subjects with additional information on the importance of cord blood donations (“Information”), (b) asking the subjects to express their intention to donate cord blood or not upon delivery (“Prompted choice”), and (c) reminding subjects about their stated intention and offering them the possibility to revise their original decision (“Multiple ask”). We took advantage of the fact that women who plan to give birth at BH visit the clinic on at least two occasions as described above—around the 12^th^ and then again at the 36–38^th^ week of gestation. The treatment conditions, to which we randomly assigned the subjects, were as follows:

- *T0: Control*. Subjects in this group did not receive any additional information. Outcomes from this group therefore provide a baseline.
- *T1: Information—first trimester*. Around the 12^th^ week of their pregnancy, subjects in this group were provided with a one-page flyer containing information about the importance of donating cord blood and its current and potential future uses.
- *T2: Prompted choice—first trimester*. Around the 12^th^ week of their pregnancy, in addition to receiving the information flyer, subjects in this group were asked to state their intention to donate cord blood upon delivery in a document with two check boxes. The first box was next to the sentence “I intend to donate the cord blood at the time of delivery, and I will request and submit the consent forms”; the second box was next to the sentence “I prefer to not make a decision at this time”. Note that checking the box was only a “soft” commitment; interested women had then to request and complete the necessary consent and health history forms to be considered for donation (see Section 2 of the Supplementary Material).
- *T3: Information—third trimester*. This condition included the same material as T1, but women would receive it around the 36–38^th^ week of pregnancy at an end-of-pregnancy ambulatory facility.
- *T4: Prompted choice—third trimester*. Women in this condition received the same treatment as those in T2, but around the 36–38^th^ week of pregnancy at the end-of-pregnancy ambulatory.
- *T5: Prompted choice first trimester, reminder and “second ask” in third trimester*. Subjects in this group were provided the same treatment in the first trimester as in T2. In addition, around the 36–38^th^ week of pregnancy at the end-of-pregnancy ambulatory facility, they were given the opportunity to confirm or revise their choice. Specifically, they were presented with a document that (a) asked them to recall their previous choice, and (b) offered them the possibility of confirming or revising their initial choice by marking one of three options: (b1) “I intend to donate the cord blood and I have already filled and handed in the consent forms,” (b2) “I intend to donate the cord blood, and I will fill and hand in the consent forms,” and (b3) “I prefer not to make a decision at this time”. (See Section 2 in the Supplementary Material). Asking more than two times might be perceived as intrusive and a source of undue influence, but we believe, based on previous research, that with only two inquiries, this risk is minimal^11^.

We limited the sample to women who came to the BH “low-risk” outpatient clinic and who were fluent in Italian, to ensure that the patients would fully understand the intervention material and did not suffer from previously diagnosed pathologies that would make them ineligible to donate the cord blood. Early pregnancy visits and blood tests were available only on Mondays, Tuesdays and Thursdays, whereas later pregnancy visits were could be scheduled all business days. Further, we limited the sample to women who reached the term of their pregnancy by the end of the study period (June 9, 2016). This sample includes women who had, by the end of the study period, reached the end of their pregnancy term, which we fixed at 42 weeks. Our assumption is that all women included in the study had the same opportunities to request, fill, and submit the consent forms, regardless of whether we treated them early or late in their pregnancy. Of the 851 women originally in the sample, 850 met this criterion.

We also administered a questionnaire to collect demographic and socio-economic information as well as measures of risk aversion, pro-social attitudes, and interest in health and medical issues. Socio-demographic questions included educational attainment, marital status, and job status. We also asked the respondents to rate their willingness to choose a low-risk, low-return opportunity over a high-risk, high-return one (1 to 5 scale), to state their interest in health-related and medical information (again on a 1 to 5 scale), as well as their interest in participating in a lottery that would give 3 prizes of 100 euros each and, if they won, how much of that sum they would commit to donating to a charity of their choice. The latter measures are meant to provide proxies for risk preferences and degree of altruism. In addition to these measures, administrative data obtained from the clinic included the participants’ year and place of birth, as well as the number of pregnancies (including the current one) and previous deliveries.

### Implementation

The clinics’ nurses administered the treatments prior to the participants seeing the physician for their routine visits. All subjects received the questionnaire. In all conditions, women completed the survey after reading the materials pertaining to their specific treatment. Three obstetric nurses were responsible for collecting the data.

We conducted the intervention on 850 pregnant women who visited BH between September 2014 and early June 2016 and satisfied the inclusion criteria. Of the 850 women included in our study, 688 (81%) gave birth at BH. As shown in Table S1 (Supplementary Material), this attrition was not significantly correlated with participant characteristics (including age, education, number of previous pregnancies, and number of previous deliveries). The only predictor of attrition is being approached for our study in the 1^st^ trimester. This is not surprising because the vast majority of women who experience miscarriages and other events that result in an unsuccessful pregnancy face these events relatively early in their pregnancy. Moreover, conversations with BH personnel revealed that a share of women who visit the hospital’s OB-GYN unit at the beginning of their pregnancy end up delivering somewhere else.

Table 3 shows the sample sizes by condition. We stopped the recruitment of women in the 1st trimester of pregnancy a few months before the end of the overall intervention to guarantee enough time for these participants to give birth by the end of the study; this procedure (in addition to the attrition described above) resulted in a smaller number of 1^st^ trimester participants. Attrition also explains the smaller number of subjects in group T5; of the 63 women originally in T5, 35 came back to BH for their late-term visit and were thus given a second chance to express their cord blood donation decision.

**Table 3:** Sample sizes, by experimental condition.

	All women in the study	Women who delivered at Buzzi hospital
Control	217	183
Info, 1st trimester	64	34
Info + Prompted choice, 1st trimester	88	45
Info, 3rd trimester	197	179
Info + Prompted choice, 3rd trimester	249	219
Info + Prompted choice, 1st + 3rd trimester	35	28
Total	850	688

Attrition and sample size imbalance, however, did not result in an unbalanced sample in terms of average characteristics of the population in each condition, with the only (obvious) exception of women recruited in the last trimester being slightly older than the others. Table S2 (in the Supplementary Material) reports statistics on the socio-demographic characteristics of the full sample of participants. On average, the women in our sample were 34 years old, had had 1.74 pregnancies (including the current one), and delivered 0.44 babies; 59% of them had a college degree or a higher level of education. Table S3 in the Supplementary Material reports the same information limited to the sample of women who delivered at BH, showing that this subgroup had very similar characteristics to the full sample. Of course, we recommend caution when interpreting the findings for T5 because of the sample size; however, as we will see below, outcomes for this group were quite striking.

We consider the following outcomes:

1. Whether participants in the three “prompted choice” conditions (T2, T4 and T5) indicated that they intended to donate the cord blood to the local public bank, when asked (in written but non-binding form).
2. Whether participants requested the consent and medical forms needed to express consent to donate and to report the parents’ health history.
3. Whether participants handed back completed and signed donation and health history forms.
4. Whether participants handed back completed donation and health history forms and were eligible to donate.
5. Whether participants who expressed their consent to donate and were eligible were subsequently unable to donate successfully, and for what reason.
6. Whether participants donated their umbilical cord blood successfully.

Outcomes 1 and 2 represent expressions of interest for donating cord blood whereas outcome 3 is a direct indication of the expecting parents’ intentions to donate. These three measures, although not representing the outcome that is ultimately most relevant (i.e., actual donations), relate more directly to our interventions because they are under the control of the study subjects. As such, by measuring these outcomes (particularly outcome 3) we are able to assess the behavioral impact of the intervention. Actual eligibility and cord blood extraction (outcomes 4, 5 and 6) depend not only on intention and consent, but also on health conditions of which women might not be aware when making their decision, as well as on events that may occur during the delivery that make the donation unfeasible. These events fall in two classes; first, medical complications during delivery imply priority for the mother and the newborn over additional procedures. Typically, cord blood collection does not occur when there are complications during delivery. Second, an unsuccessful donation may result from organizational and institutional issues related to the extraction and collection process. Our ability to measure missed donations due to organizational and institutional constraints—one of the most novel aspects of our study—allows us to quantify the relative impact of institutional structures and rules with respect to the effect of behavioral interventions *per se*. In particular, we are able to assess what the impact of our interventions would have been in the absence of organizational or institutional impediments.

### Trial registration and sample

The registration of the protocol (#0000695) is available at the registry of randomized controlled trials of the American Economic Association (https://www.socialscienceregistry.org/). We registered the trial on April 15^th^ 2015, and in the registration we report the starting data collection as November 1^st^ 2014. We also ran a pilot trial in the months of September and October 2014. We were expecting to collect 1,800 observations, but we eventually had access to only 850 women, including the pilot period as well as the full period before registration. Because we did not modify the design before and after the registration, or before and after the pilot, and because of the smaller sample size, we decided to perform our analyses on the full set of available data. Limiting the sample to the period from November 1^st^ 2014 or from April 15^th^ 2015 yields qualitatively similar results, although with slightly larger standard errors. The results from these subsamples are available from the authors upon request. Although the end date indicated in the registered protocol is May 31^st^ 2016, we included one woman who was recruited before that date and delivered on June 9^th^.

## Electronic supplementary material

Supplementary Material
