# Supplementary material for: Motivating Cord Blood Donation with Information and Behavioral Nudges
Source: Sci Rep. 2018 Jan 10;8:252. doi: 10.1038/s41598-017-18679-y (PMC5762860; doi:10.1038/s41598-017-18679-y)
Supplement: Supplementary file 1 — Supplementary Material [file 41598_2017_18679_MOESM1_ESM.pdf]

SUPPLEMENTARY MATERIAL

Motivating Cord Blood Donation with Information and Behavioral Nudges

Daniela Grieco
Nicola Lacetera
Mario Macis
Daniela Di Martino

1. Additional notes to the text

We provide additional details and considerations about umbilical cord blood donation in general and in the Italian context. We also describe as well as of the broader literature about motivations for altruistic behavior and the donation of body parts and fluids in particular.

- A concern that recently emerged with regard to donating cord blood is that the procedure requires a relatively fast clamping of the umbilical cord, thus potentially limiting the flow of blood from the mother to the baby right after delivery (Focosi 2014). Some evidence indicates that delayed cord clamping decreases iron deficiency infants (Ceriani et al. 2010; Chaparro et al. 2006). However, there is no specific evidence of negative effects on healthy full-term babies, and the time frame for clamping to collect a sufficient amount of cord blood (one to three minutes) is consistent with WHO prescription about the optimal time of clamping in general (http://www.who.int/elena/titles/cord_clamping/en/)
- The likelihood that a child will need her/his own stem cells for treatment in their early years is very small, between 1/5000 and 1/20,000 (Weeks 2012). The American and Canadian Colleges of Obstetricians and Gynecologists as well as the American Academy of Pediatrics, for example, recommend that altruistic donations to public banks should be encouraged, and do not recommend self-storage.
- These are the requirements, according to Italian law, to allow for self donation/storage: a) the child must have pathologies that require the use of stem cells, or the mother's and father's families have shown a high risk of having children with genetically determined pathologies; b) the only pathologies considered for cord blood "self" donation are those listed in the 18/11/2009 Decree; c) the request of storage must be authorized by a doctor specialized in any of the admitted pathologies; d) in case of other diseases, the authorizing doctor must "provide scientific evidence of any effect of the cure using cord blood stem cells".
- Despite the lack of reliable scientific evidence and the legal ban, more than 60,000 Italian cord blood units from Italian babies are currently conserved in foreign private banks; not a single unit has been used to date (Contu 2011). The 18/11/2009 Decree recognizes that there is no scientific evidence showing any benefit from the self-storage (and use) of cord blood. Moreover, it states that the self-storage is against the principles of "equality of treatment and opportunities" of the Italian legal system. Italy and France are the only countries in the European Union where self-storage is not allowed on domestic territory (in fact, France does not even allow the "storage abroad" option). In the other EU countries, banks for self-storage are allowed, although the European Commission in 2005 explicitly took a position against self-storage, recommending member states not to allow the constitution of private cord blood banks. Furthermore, in 2011 the Italian Antitrust Commission forced six foreign private banks to clarify the information that they were diffusing on the real therapeutic advantages of self-storage, on the number of transplants that actually involved self-stored blood versus donations, and on the limited time-span of conservation (15 years instead of the advertised 25 years).

Cited references

Ceriani Cernadas, JM, Carroli, G, Pellegrini, L et al. The effect of early and delayed umbilical cord clamping on ferritin levels in term infants at six months of life: a randomized, controlled trial. ((In Spanish).)Arch Argent Pediatr. 2010; 108: 201–208

Chaparro, CM, Neufeld, LM, Tena Alavez, G, Eguia-Líz Cedillo, R, and Dewey, KG. Effect of timing of umbilical cord clamping on iron status in Mexican infants: a randomized controlled trial. Lancet. 2006; 367: 1997–2004

Contu, L. (2011) "Il Dibattito Sul Sangue del Cordone Ombelicale: A Chi e a Che Cosa Serve la Conservazione Privata del Sangue Cordonale?" ADOCES.

Focosi, D., 2014. Delayed cord clamping. *The Lancet*, *384*(9955), p.1668.

Weeks, C. (2012). "Why Banking on Cord Blood Isn't Necessarily a Good Idea," *The Globe and Mail*, May 26.

2. Text for information flier and prompted choice survey

We report below the text that we used for the information flyer common to conditions T1 trough T5. The flyer, elaborated by the authors after consultations with the hospital personnel and approved by the ethics board as described in the main text, was in Italian. We report, in italics, the English translation.

Hai mai considerato di donare il cordone ombelicale dopo la nascita del tuo bambino?

Il cordone ombelicale contiene sangue ricco di cellule staminali, le stesse del midollo osseo, che sono in grado di generare tutte le cellule del sangue e del sistema immunitario.

Questo sangue può essere utilizzato per il trapianto come il midollo osseo, salvando così chi ha un tumore del sangue come la leucemia.

Per queste persone, spesso bambini, il trapianto di queste cellule, è l'unica speranza di vita.

Il prelievo del sangue cordonale avviene dopo il parto, senza nessun rischio per la mamma e per il neonato.

Per maggiori informazioni, e per il materiale dedicato alla donazione, la preghiamo di rivelgersi al personale degli ambulatori o di inviare una email a infodonazione@gmail.com.

Have you considered donating the umbilical cord after the birth of your child?

The umbilical cords contain blood that is rich of stem cells, the same that are present in the bone marrow, which are able to generate all cells in the blood and the immune system.

This blood can be used for transplants just like the bone marrow, thus saving those who suffer from blood cancers such as leukemia.

For these patients, often children, the transplant of these cells is often the only hope to survive their disease.

The collection of the umbilical cord blood occurs after delivery, with no risks for the mother or the newborn.

For more information and for material related to the donation, please ask the outpatient clinic personnel or send an email to infodonazione@gmail.com.

Here is the text that we used for the prompted choice survey in conditions T2 (first trimester), T4 (third trimester) and T5 (first trimester only). The text, elaborated by the authors after consultations with the hospital personnel and approved by the ethics board as described in the main text, was in Italian. We report, in italics, the English translation.

Alla luce delle informazioni ricevute, sarebbe interessata alla donazione del sangue cordonale?

a□ Sì, sono interessata a donare e intendo ritirare e compilare i moduli per il consenso il prima possibile

b□ Preferisco non prendere una decisione in questo momento

Nel caso Lei sia interessata a ricevere ulteriore materiale informativo sulla donazione del cordone ombelicale e a ritirare i moduli per il consenso formale alla donazione, per favore si rivolga al personale.

La ringraziamo della Sua disponibilità.

Based on the information received, would you be interested in donating the umbilical cord blood?

a□ Yes, I am interested and I intend to pick up and fill the consent forms as soon as possible

b□ I prefer to not make a decision at this time

If you are interested in receiving additional information on the donation of cord blood and in taking the documents for the formal consent to donate, please ask the clinic's personnel.

Thank you for your interest.

Below is the text of the second prompted choice survey for women in condition T5 (third trimester). The text, elaborated by the authors after consultations with the hospital personnel and approved by the ethics board as described in the main text, was in Italian. We report, in italics, the English translation.

Quando Le è stato chiesto in precedenza riguardo alla Sua intenzione di donare il sangue cordonale, Lei ha dichiarato che (indichi l'opzione che si riferisce a Lei):

a□ Intendeva donare il sangue del cordone ombelicale

b□ Preferiva non prendere una decisione in quel momento

La preghiamo ora di selezionare l'opzione che La rappresenta meglio oggi:

a□ Intendo donare il sangue del cordone ombelicale e ho già compilato e consegnato i moduli per il consenso

b□ Intendo donare il sangue del cordone ombelicale e compilerò e consegnerò i moduli per il consenso al più presto

c□ Preferisco non prendere una decisione in questo momento

Nel caso Lei sia interessata a ricevere ulteriore materiale informativo sulla donazione del cordone ombelicale e a ritirare i moduli per il consenso formale alla donazione, per favore si rivolga al personale.

When you were previously asked about your intention to donate the umbilical cord blood, you stated that (please check the option that applies to you):

a□ You intended to donate the umbilical cord blood

b□ You preferred to not make a decision at that time

Please select the option that represents you best as of today:

a□ I intend to donate the umbilical cord blood and I have already filled and handed in the consent forms

b□ I intend to donate the umbilical cord blood and will fill and hand in the consent forms as soon as possible

c□ I prefer to not make a decision at this time

If you are interested in receiving additional information on the donation of cord blood and in taking the documents for the formal consent to donate, please ask the clinic's personnel.

Thank you for your interest.

3. Supplementary tables

Table S1: Attrition from the sample.

	(1)	(2)
Info, 1st trimester	0.312**	0.289**
	(0.052)	(0.053)
Info+Prompted choice, 1st trimester	0.332**	0.331**
	(0.047)	(0.046)
Info, 3rd trimester	-0.065+	-0.049
	(0.036)	(0.036)
Info+Prompted choice, 3rd trimester	-0.036	-0.035
	(0.034)	(0.034)
Info+Prompted choice, 1st+3rd trimester	0.043	0.089
	(0.067)	(0.064)
Age 34+		-0.009
		(0.027)
First pregnancy		-0.066+
		(0.038)
Previous delivery		0.050
		(0.039)
Foreign born		-0.060+
		(0.035)
College degree		-0.039
		(0.027)
Married		-0.013
		(0.026)
Preference for high risk-high return vs low risk-low return (1-5)		0.020
		(0.015)
Interest toward medical info (1-5)		-0.009
		(0.012)
Willingness to participate in lottery		0.031
		(0.044)
Amount to charity if win lottery		-0.019
		(0.034)
Observations	850	767
R-squared	0.123	0.143

Notes: Ordinary least squares regressions. The dependent variable is equal to 1 for women who were included in the study but did not deliver at the Buzzi hospital, and for whom we do not have complete outcomes. Standard errors are in parentheses. ** p<0.01, * p<0.05, + p<0.1.

Table S2: Averages of main variables, by experimental condition, and balance checks.

Full Sample.

	Overall average	Control	Info, 1st trimester	Info+Prompted choice, 1st trimester	Info, 3rd trimester	Info+Prompted choice, 3rd trimester	Info+Prompted choice, 1st+3rd trimester
% age 34+	50.1%	53.0%	59.4%	60.2%	44.1%	44.1%	60.0%
% first pregnancy	49.8%	54.0%	52.5%	52.9%	48.4%	45.8%	48.6%
% first delivery	63.9%	64.9%	65.0%	67.1%	67.4%	59.3%	60.0%
% foreign born	16.8%	20.9%	31.1%	16.3%	12.2%*	13.7%*	14.7%
% with college degree	59.1%	56.9%	51.6%	45.5%	61.9%	65.7%	57.1%
% married	56.7%	53.2%	56.3%	54.0%	55.8%	59.3%	71.4%*
Preference for high risk-high return vs low risk-low return (1-5)	2.79	2.88	2.68	2.83	2.84	2.73	2.57*
Interest toward medical info (1-5)	3.39	3.30	3.65	3.38	3.42	3.40	3.26
% willing to participate in the lottery	86.4%	87.5%	73.4%*	87.4%	90.3%	86.3%	80.0%
average amount given to charity if win lottery (as % of total)	56.7%	54.2%	41.6%*	56.4%	58.6%	61.0%	60.0%
N.	850	217	64	88	197	249	35

Notes: Asterisks indicate statistically significant differences from the control group ($p<0.05$).

Table S3: Averages of main variables, by experimental condition, and balance checks.

Sample limited to women who delivered at the Buzzi hospital.

	Overall average	Control	Info, 1st trimester	Info+Prompted choice, 1st trimester	Info, 3rd trimester	Info+Prompted choice, 3rd trimester	Info+Prompted choice, 1st+3rd trimester
% age 34+	51.5%	50.8%	64.7%	66.7%	42.4%	44.2%	57.1%
% first pregnancy	50.1%	53.4%	55.9%	57.8%	48.3%	46.5%	50.0%
% first delivery	63.6%	64.0%	70.6%	66.7%	65.9%	59.9%	60.7%
% foreign born	16.4%	20.9%	33.3%	18.6%	12.3%*	13.2%*	14.8%
% with college degree	62.0%	59.3%	50.0%	46.7%	63.7%	67.9%	60.7%
% married	57.0%	54.9%	52.9%	48.9%	57.0%	59.2%	71.4%
Preference for high risk-high return vs low risk-low return (1-5)	2.78	2.90	2.65	2.78	2.86	2.67*	2.50*
Interest toward medical info (1-5)	3.41	3.30	3.97*	3.40	3.42	3.43	3.21
% willing to participate in the lottery	87.0%	86.8%	67.6%*	84.4%	91.5%	87.6%	82.1%
average amount given to charity if win lottery (as % of total)	57.7%	54.3%	41.5%	49.9%	59.7%	62.2%	65.0%
N.	688	183	34	45	179	219	28

Notes: Asterisks indicate statistically significant differences ($p<0.05$) from the control group.

Table S4: Regressions results, with control variables. Full sample.

	(1)	(2)	(3)	(4)	(5)
**Outcome variable**	Expressed intention to donate	Requested donation info and consent forms	Handed in signed donation consent forms	Handed in signed donation consent forms, and eligible to donate	Actual donation
Info, 1st trimester		0.060	0.065	0.009	0.035
		(0.069)	(0.070)	(0.066)	(0.040)
Info + Prompted choice, 1st trimester		0.352**	-0.011	0.001	-0.014
		(0.060)	(0.060)	(0.056)	(0.034)
Info, 3rd trimester		0.342**	0.277**	0.188**	0.060*
		(0.047)	(0.047)	(0.044)	(0.027)
Info + Prompted choice, 3rd trimester	0.066	0.496**	0.312**	0.187**	0.069**
	(0.058)	(0.044)	(0.045)	(0.042)	(0.025)
Info + Prompted choice, Multiple ask	0.303**	0.503**	0.285**	0.264**	0.133**
	(0.089)	(0.084)	(0.084)	(0.079)	(0.048)
Age 34+	-0.056	0.047	0.071*	0.037	0.042*
	(0.050)	(0.035)	(0.035)	(0.033)	(0.020)
First pregnancy	-0.041	-0.035	-0.047	0.041	0.013
	(0.073)	(0.049)	(0.050)	(0.047)	(0.028)
First delivery	0.107	0.086+	0.163**	0.077	0.009
	(0.075)	(0.051)	(0.052)	(0.048)	(0.029)
Foreign born	-0.012	-0.095*	-0.051	-0.072+	-0.031
	(0.069)	(0.045)	(0.045)	(0.042)	(0.026)
College degree	0.093+	0.078*	0.085*	0.087**	0.000
	(0.051)	(0.035)	(0.035)	(0.033)	(0.020)
Married	-0.089+	-0.016	0.013	0.021	0.020
	(0.049)	(0.033)	(0.034)	(0.032)	(0.019)
Preference for high risk-high return vs low risk-low return (1-5)	0.036	0.003	-0.004	-0.002	-0.002
	(0.028)	(0.019)	(0.019)	(0.018)	(0.011)
Interest toward medical info (1-5)	0.008	-0.002	0.008	0.007	0.005
	(0.023)	(0.015)	(0.015)	(0.014)	(0.009)
Willingness to participate in lottery	-0.041	-0.140*	-0.119*	-0.087	-0.157**
	(0.082)	(0.057)	(0.057)	(0.054)	(0.032)
Amount to charity if win lottery	0.219**	0.136**	0.075+	0.026	0.040
	(0.066)	(0.044)	(0.044)	(0.042)	(0.025)
Constant	0.413**	0.220*	0.065	0.047	0.092+
	(0.143)	(0.095)	(0.095)	(0.090)	(0.054)
N. of observations	335	767	767	767	767
R squared	0.106	0.216	0.144	0.092	0.067

Notes: Ordinary least squares regressions. Standard errors are in parentheses. ** p<0.01, * p<0.05, + p<0.1.

Table S5: Regressions results, with control variables. Women who delivered at BH.

Outcome variable	(1) Expressed intention to donate	(2) Requested donation info and consent forms	(3) Handed in signed donation consent forms	(4) Handed in signed donation consent forms, and eligible to donate	(5) Handed in signed donation consent forms, eligible to donate, and no complications during delivery	(6) Actual donation
Info, 1st trimester		0.180*	0.198*	0.085	0.103	0.071
		(0.087)	(0.091)	(0.086)	(0.070)	(0.053)
Info + Prompted choice, 1st trimester		0.400**	0.116	0.105	0.026	-0.006
		(0.076)	(0.080)	(0.076)	(0.061)	(0.047)
Info, 3rd trimester		0.305**	0.243**	0.162**	0.111**	0.067*
		(0.049)	(0.052)	(0.049)	(0.039)	(0.030)
Info + Prompted choice, 3rd trimester	0.009	0.482**	0.302**	0.185**	0.123**	0.076**
	(0.072)	(0.047)	(0.049)	(0.046)	(0.038)	(0.029)
Info + Prompted choice, Multiple ask	0.291**	0.525**	0.382**	0.352**	0.175*	0.174**
	(0.104)	(0.093)	(0.098)	(0.092)	(0.075)	(0.057)
Age 34+	-0.050	0.038	0.086*	0.051	0.060*	0.050*
	(0.054)	(0.038)	(0.040)	(0.038)	(0.030)	(0.023)
First pregnancy	-0.099	-0.061	-0.045	0.068	0.105*	0.017
	(0.083)	(0.055)	(0.058)	(0.055)	(0.044)	(0.034)
First delivery	0.160+	0.103+	0.172**	0.058	-0.048	0.006
	(0.083)	(0.057)	(0.060)	(0.056)	(0.046)	(0.035)
Foreign born	-0.017	-0.134**	-0.082	-0.087+	-0.060	-0.045
	(0.072)	(0.048)	(0.051)	(0.048)	(0.039)	(0.030)
College degree	0.102+	0.089*	0.071+	0.096*	0.029	-0.002
	(0.055)	(0.038)	(0.040)	(0.038)	(0.030)	(0.023)
Married	-0.077	-0.017	0.010	0.023	0.039	0.025
	(0.053)	(0.036)	(0.038)	(0.036)	(0.029)	(0.022)
Preference for high risk-high return vs low risk-low return (1-5)	0.025	0.000	0.009	0.007	0.001	-0.001
	(0.030)	(0.021)	(0.022)	(0.021)	(0.017)	(0.013)
Interest toward medical info (1-5)	0.006	-0.004	0.009	0.009	0.007	0.006
	(0.024)	(0.017)	(0.017)	(0.016)	(0.013)	(0.010)
Willingness to participate in lottery	-0.046	-0.130*	-0.111+	-0.090	-0.129*	-0.192**
	(0.088)	(0.062)	(0.065)	(0.062)	(0.050)	(0.038)
Amount to charity if win lottery	0.254**	0.140**	0.078	0.020	0.018	0.044
	(0.070)	(0.048)	(0.050)	(0.048)	(0.039)	(0.030)
Constant	0.475**	0.250*	0.042	0.028	0.065	0.116+
	(0.154)	(0.102)	(0.107)	(0.102)	(0.082)	(0.063)
N. of observations	272	644	644	644	644	644
R squared	0.133	0.214	0.121	0.084	0.060	0.078

Notes: Ordinary least squares regressions. Standard errors are in parentheses. ** $p<0.01$, * $p<0.05$, + $p<0.1$.
